# Broomcorn and foxtail millet were cultivated in Taiwan about 5000 years ago

**DOI:** 10.1186/s40529-016-0158-2

**Published:** 2017-01-02

**Authors:** Chen-Hwa Tsang, Kuang-Ti Li, Tze-Fu Hsu, Yuan-Ching Tsai, Po-Hsuan Fang, Yue-Ie Caroline Hsing

**Affiliations:** 1 Institute of History and Philosophy, Academia Sinica, Taipei, Taiwan; 2 Institute of Plant and Microbial Biology, Academia Sinica, Taipei, Taiwan; 3 Department of Agronomy, National Taiwan University, Taipei, Taiwan

**Keywords:** Archaeobotany, Broomcorn millet, Foxtail millet, Yellow foxtail

## Abstract

**Background:**

Archaeobotanical remains of millet were found at the Nan-kuan-li East site in Tainan Science Park, southern Taiwan. This site, dated around 5000–4300 BP, is characterized by remains of the Tapenkeng culture, the earliest Neolithic culture found so far in Taiwan. A large number of millet-like carbonized and charred seeds with varied sizes and shapes were unearthed from the site by the flotation method. Since no millet grain was ever found archaeologically in Taiwan previously, this discovery is of great importance and significance. This paper is in an attempt to further analyze these plant remains for a clearer understanding of the agricultural practice of the ancient inhabitants of the Nan-kuan-li East site.

**Result:**

We used light and scanning electron microscopy to examine the morphological features of some modern domesticated and unearthed seeds to compare and identify the archaeobotanical remains by three criteria: caryopsis shape, embryo notch, and morphology of lemma and palea. We also developed a new methodology for distinguishing the excavated foxtail and broomcorn millet seeds.

**Conclusion:**

Two domesticated millet, including broomcorn millet (*Panicum miliaceum*) and foxtail millet (*Setaria italica*), as well as one wild millet species, yellow foxtail (*Setaria glauca*), were identified in the unearthed seeds. Together with the millet remains, rice was also cultivated in the area. Archaeological evidence shows that millet and rice farming may have been important food sources for people living about 5000 years ago in southern Taiwan.

## Background

**Fig. 1:**
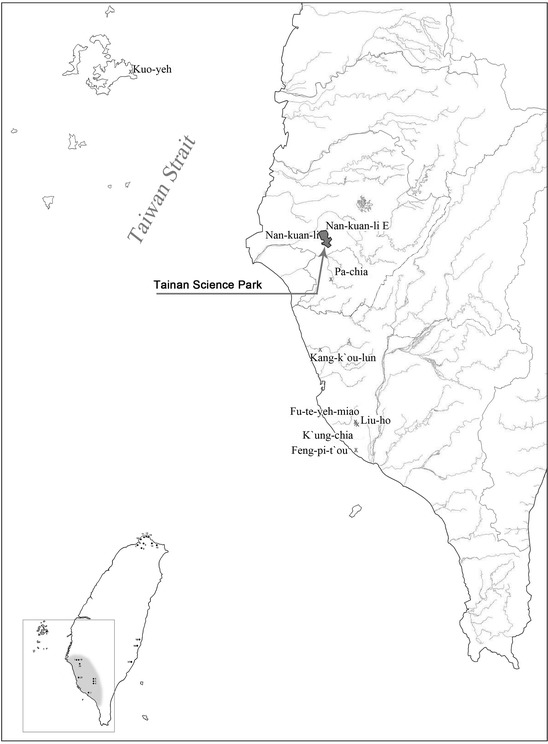
The location of Nan-kuan-Li (NKL) and Nan-kuan-Li East site. The insert is Taiwan and the nearby islands. The location of the Tainan Science Park, NKL and NKLE are indicated in the zoom-in image

Plant remains collected from archaeological studies at excavated sites in Taiwan revealed the existence of both millet and rice grains. For instance, during the construction of Tainan Science Park in Tainan county, a Neolithic site was found on the floodplain of Hsin-shih Hsiang, Tainan county (Fig. 1a) and was discovered because of the construction of a factory building, named Nan-kuan-li East (NKLE; spelling also as Nanguanli East) (Tsang 2005; Tsang et al. 2006; Tsang 2013; Li 2013). The archaeological project, led by Cheng-hwa Tsang, conducted a salvage excavation at the site from September 2002 to March 2003 (Fig. 1b). An area of about 2400 m^2^ was unearthed. Most of the excavated area exhibited a rich deposit of archaeological remains and two clearly stratified cultural components. However, only a small part of the area had three clearly stratified cultural layers. Each of the layers was approximately 20–30 cm thick. Forty-nine radiocarbon dates have been obtained from the NKLE East site. All of the dates have been calibrated, with mean range from 5000 to 4300 cal BP and no clear age difference between the three layers. Therefore, these layers are probably a local depositional phenomenon at the site and have no clear chronological significance (Tsang and Li 2015). They are the earliest large-scale seed remains found in Taiwan up to now. Archaeological remains from NKLE are extremely abundant, including pottery, stone tools, shell and bone tools as well as animal bones, plant remains and human burials (Tsang et al. 2006; Li 2013; Tsang and Li 2015). These cultural remains basically bear the characteristics of the Tapenkeng culture, the earliest Neolithic culture found so far in Taiwan.

The discovery of a large number of carbonized and charred millet grains from the NKLE site is especially noteworthy (Tsang and Li 2015). Because no earlier millet grains were ever found archaeologically in Taiwan, this discovery is apparently of great importance and significance, assuming that prehistoric people in Taiwan at 5000 BP probably already knew how to grow these plants, and the mode of their subsistence was no longer limited to hunting and gathering. This assumption seems to gain its support not only from direct evidence of millet grains and indirect evidence such as the types of tools used for agricultural activities at that time. The most distinguishing tools are shell knives. The knives were formed by cutting and polishing shells that are relatively flat and thin. On comparison with ethnographical records, these shell knives were probably used as a kind of grain-harvesting tool (Tsang and Li 2015).

The species of millet grains have not been genetically identified. However, they look similar to foxtail millet (*Setaria italica* L.), which is still cultivated by Austronesian-speaking people in Taiwan. To understand the agricultural practice of the ancient inhabitants of this site, this study aimed at a detailed analysis of the morphological characteristics of the millet grains and proper identification of their taxon.

In addition to millet, the plant remains collected from archaeological studies at unearthed sites in Taiwan revealed the existence of rice grains (Tsang et al. 2006; Hsieh et al. 2011; Tsang 2012; Li 2013; Tsang and Li 2015). Because these crops were domesticated in China (Nasu et al. 2007; Lu et al. 2009), we also aimed to reveal the early agriculture products grown in Taiwan. Two types of millet, including foxtail millet and broomcorn millet (*Panicum miliaceum*, also known as Proso millet), were widely cultivated in several northern Chinese cultures by 8000 BP (Liu and Kong 2004; Lu et al. 2009; Zhang et al. 2012; Weisskopf et al. 2015). Archaeobotany study of the Baligang site in north central China revealed mixed farming of rice and two kinds of millet (Weisskopf et al. 2015). We aimed to determine whether similar farming was present in Taiwan thousands of years ago.

## Methods

### Archaeological materials

More than 120,000 millet grains were collected from the NKLE (23º6′58″N, 120º16′35″E, altitude 0.5 m) site by using a floatation method. We selected about 3000 millet seeds as analytical samples from randomly chosen excavation units of the NKLE EB-Y series, including NKLE-E5T2P6L56F1, NKLE-E5T7P0L57F2, NKLE-F4T4P1L55F4, NKLE-E3T3P2L56F1-1, and NKLE-E4T1P3L56F1-1. The samples were used to estimate the ratio of large to small seeds. Some of these samples contained lemma and palea remains and were used to study the presence of different types of millet seeds. We then used 2000 complete millet seeds from NKLE-F4T3P7L57F1 for microscopy observation and morphological analysis of the angle of the embryo notch, width of the opening of the embryo notch, and depth of the embryo notch.

### Characterization of millet seeds

**Fig. 2:**
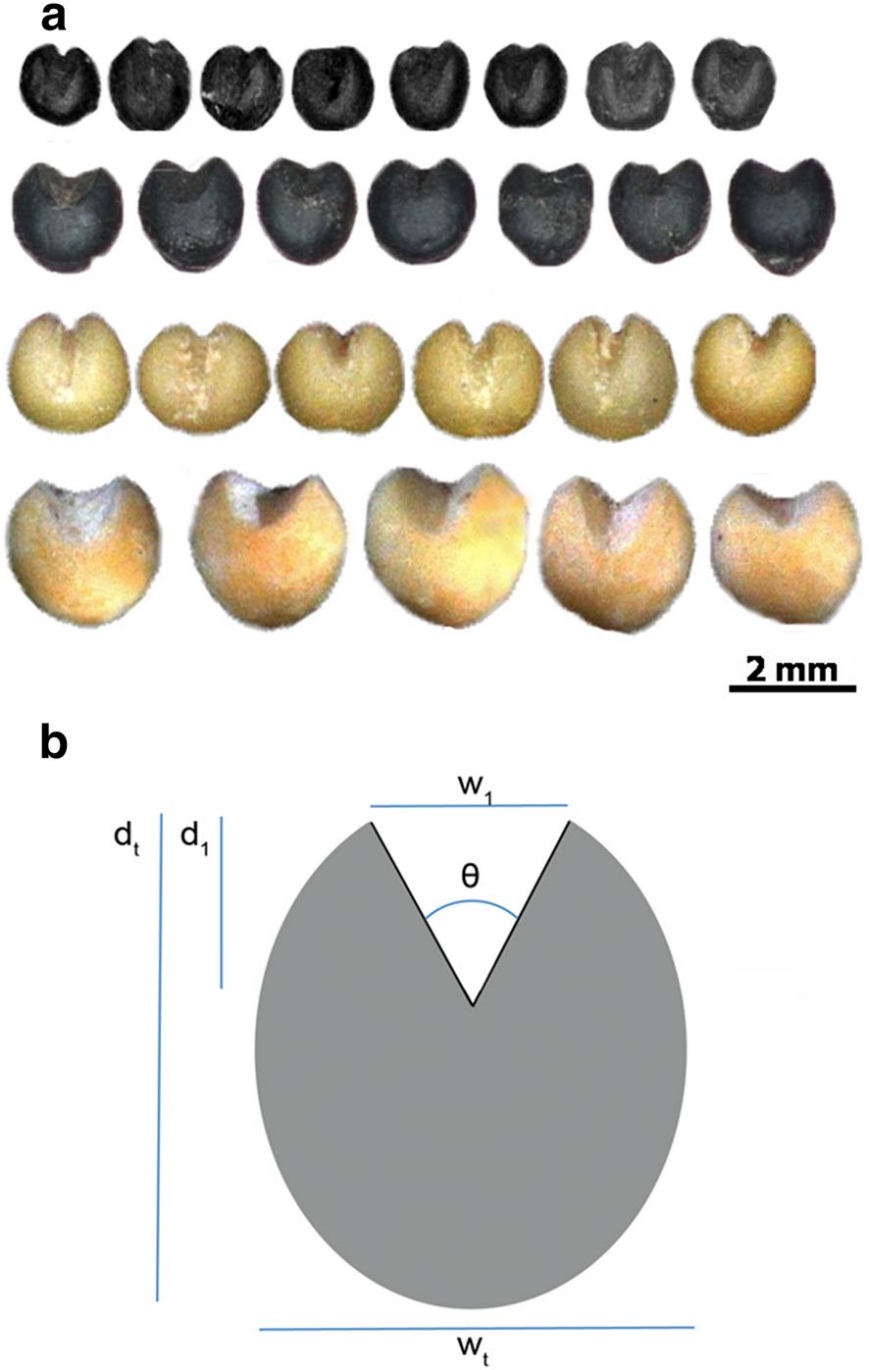
Characterization of ancient and modern millet seeds. **a** *Top row* unearthed foxtail millet seeds; *second row* de-embryonated foxtail millet seeds; *third row* unearthed broomcorn millet seeds; *fourth row* de-embryonated broomcorn millet seeds. *Scale bar* 2 mm. **b** Illustration for measurement of millet seeds. θ angle of embryo notch; *w*_*1*_ width of the opening of the embryo notch; *w*_*t*_ width of the seed at the longest side; *d*_*1*_ depth of embryo notch; *d*_*t*_ depth of the seed at the longest side

The lemma and palea of seed of modern broomcorn (*Panicum miliaceum*, collected from the Smangus Taiwan aboriginal village) and foxtail millet (collected from the Dan-lin aboriginal village) were first removed, then the embryo was removed (Fig. 2a). Photographs of the unearthed seeds and the de-embryonated modern seeds were analyzed by using ImageJ (https://imagej.nih.gov/ij/) (Schneider et al. 2012). We measured several parameters: (1) angle of the embryo notch, θ; (2) width of the opening of the embryo notch, w_1_; (3) total width of the seed, w_t_; (4) depth of the embryo notch, d_1_; and (5) depth of the seed at the longest side, d_t_ (Fig. 2b). The depth percentage of the seed was calculated as d1/dt and width percentage as w1/wt.

A digimatic caliper (Model CD-6”ASX, Mitutoyo, Japan) was used to measure the width (w), length (l) and thickness (t) of unearthed seeds. The volume of seeds was estimated by w*l*t*π*1/6. We also used a scanner to record images of the millet seeds for ImageJ analysis. The output for each seed consisted of the longest width (w) and the longest length (l). The true seed area should be calculated as w*l*0.25*π, but we used the seed area index (w*l) instead.

### Microscopy observation

**Fig. 3:**
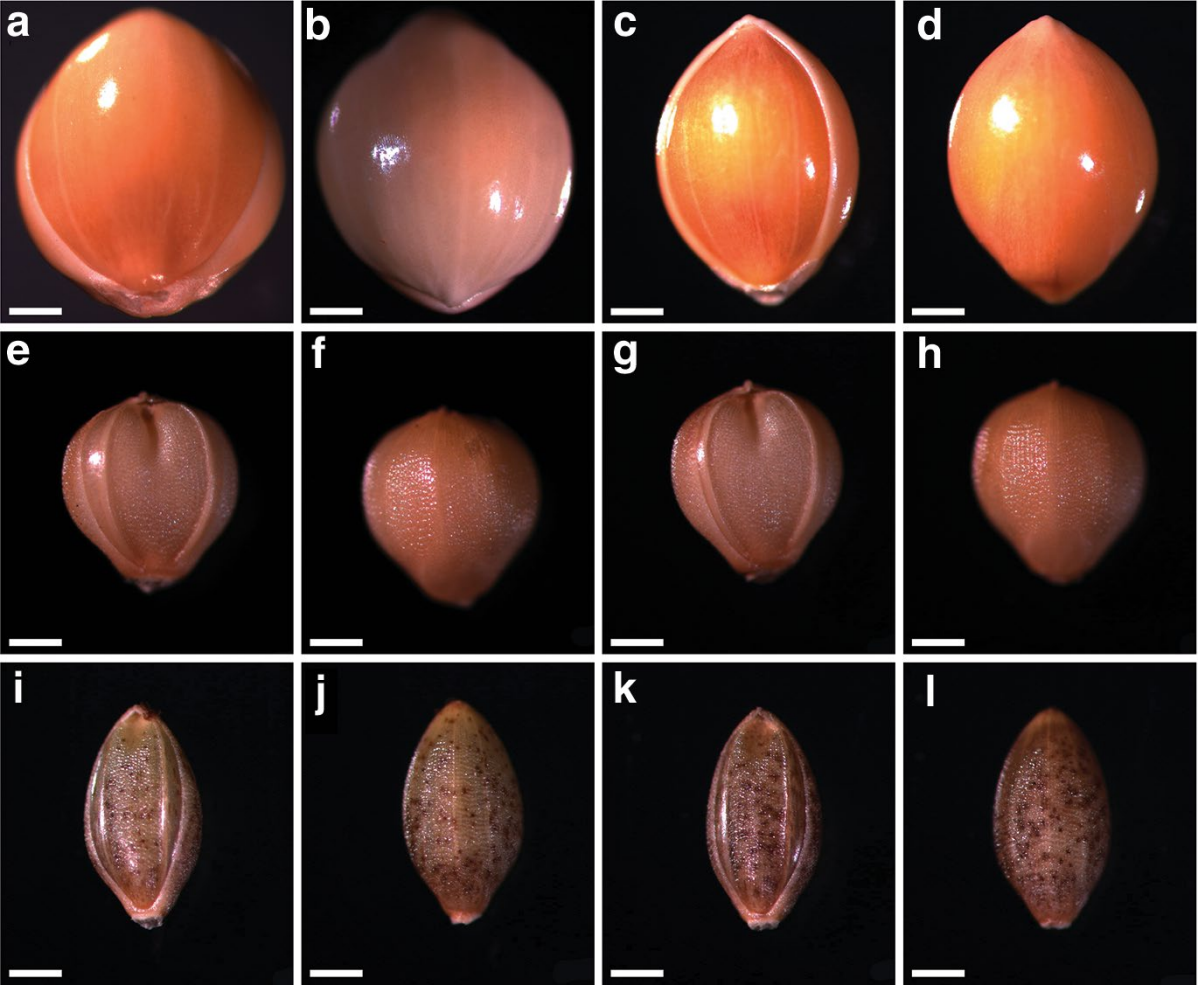
Modern millet grains. **a**, **b**, *Panicum miliaceum* seed imported from Australia; **c**, **d**, *P. miliaceum* seed from the Smangus aboriginal village, Taiwan; **e**–**h**, *Setaria italica* seeds from Paiwan aboriginal villages, Taiwan; **i**–**l**, *Setaria glauca* seeds. **a**, **c**, **e**, **g**, **i**, **k** ventral view; **b**, **d**, **f**, **h**, **j**, **l** dorsal view. *Scale bar* 0.5 mm

Previous morphological studies of unearthed foxtail millet grains (Fukunaga et al. 1997) indicated that grain shape was less affected by environmental conditions than was grain size. Thus, we mainly used grain shape to distinguish the millet species. The surface structure of the unearthed seeds and the seeds of modern broomcorn millet, foxtail millet, and yellow foxtail (*Setaria glauca*) were observed and identified. Two kinds of modern broomcorn millet were used, including those imported from Australia (purchased from a store and had no variety name, used in Fig. 3a, b) or from the Smangus Taiwan aboriginal village (Atayal tribe, used in Figs. 2a, 3c, d). For foxtail millet, we used seeds collected from plants grown in the Dan-lin aboriginal village (Paiwan tribe; used in Figs. 2a, 3e–h). The two kinds from the aboriginal villages are landraces. The seed surfaces were observed from the dorsal, ventral and lateral views by stereomicroscopy (Lumar, Zeiss), and their sizes and shapes were recorded. The surface sculpturing of the upper lemma and palea was then observed by SEM to distinguish seeds of *P. miliaceum*, *S. italica*, and *S. glauca*. The samples were placed on sample stubs, coated with gold, and observed by high-resolution SEM (Zeiss FEI Quanta 200/Quorum PP2000TR FEI).

## Results and discussion

### Both carbonized and charred seeds were present in the unearthed NKLE sites

**Fig. 4:**
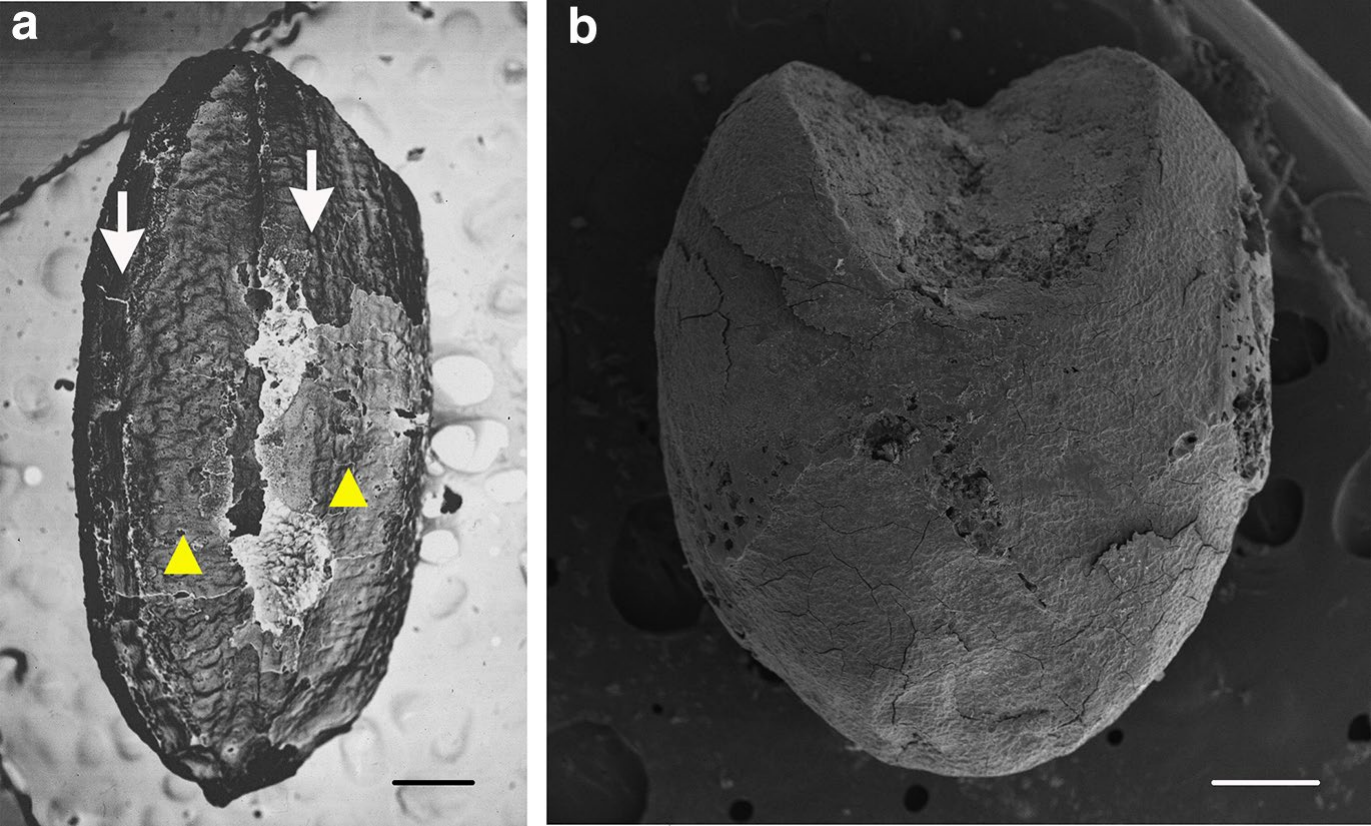
Some unearthed seeds contained bran. **a** rice grain; **b** millet grain. *Scale bars* **a** 0.5 mm; **b** 0.2 mm. *Arrow* aleurone layer; *arrowhead* naked seed

The first step was to determine whether the seeds were carbonized or charred. Because some seeds were present in trash pits and others were in living areas, we considered that those in the trash pits were charred. In addition, we also checked the NKLE seeds carefully and found that some seeds contained bran (Fig. 4). Figure 4a illustrates an unearthed rice seed, with about one-third of the surface covered with bran (also known as the aleurone layer, white arrow) and the rest was naked seed (yellow arrow head), with an empty embryo cavity (top right corner). Figure 4b illustrates an unearthed millet seed, with the whole surface covered with bran. In addition, the embryo cavity is empty (the top center). The aleurone layer is the outermost layer of the endosperm of cereal grains and is still alive in mature seeds. Next to the aleurone layer is the inner starchy endosperm, dead tissue in mature seeds. Mature rice, broomcorn millet and foxtail millet seeds contain only one cell layer of aleurone and the layers are fragile (Matsuo and Hoshikawa 1993; Zarnkow et al. 2007; Hodson and Parry 1982). We used the floatation method to collect seeds, and most of the lemma and palea were absent during the process. However, we still could detect bran covering the naked seed. Therefore, these seeds had never been burned because the aleurone layer would be distorted and destroyed by fire. Thus, we suggest that both carbonized and charred seeds were present in the unearthed sites.

### Characterization of the millet seeds at NKLE

**Fig. 5:**
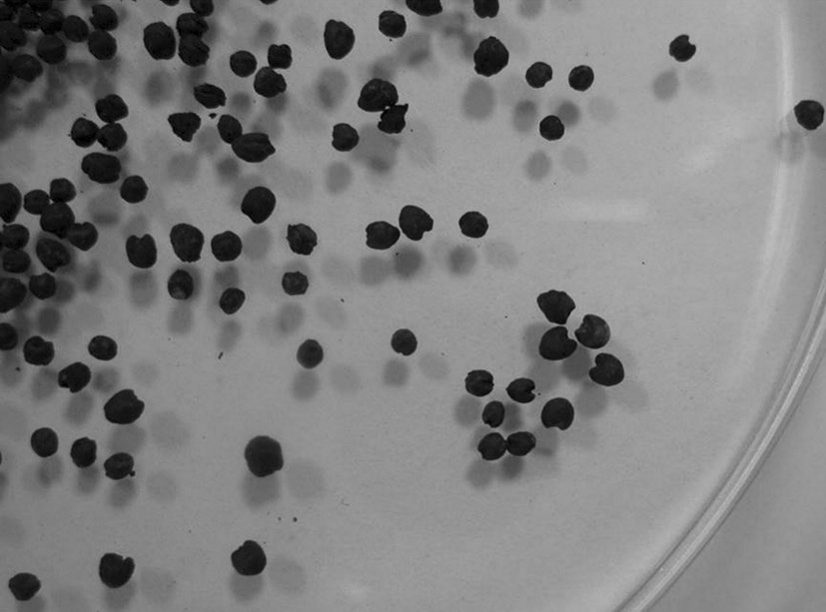
Two types of carbonized millet-like seeds present at the NKLE unearthed site in southern Taiwan. Both large and small seeds were present

After detailed analysis, the plant remains at the NKLE site were classified, with an estimated 8717 rice remains and 110,860 millet-like (small grain) remains (Tsang 2012). The sizes and shapes of the seeds in the millet varied (i.e., some seeds were larger than others). We then randomly chose five groups of samples, for a total of 3000 seeds, to check seed size (Fig. 5). The ratio of large to small seeds was 1:2.3, for more small seeds. In addition, many large seeds had a wide embryo groove. In our previous study, we found foxtail millet at the NKLE site (Hsieh et al. 2011). In the present study, we found other types of millet (i.e., *Panicum miliaceum*, broomcorn millet or proso millet) in the millet remains. As mentioned, previous studies of the morphology of unearthed rice (Matsuo 1952) and foxtail millet grains (Fukunaga et al. 1997) indicated that grain shape is less affected by environmental conditions than is grain size. Therefore, we used seed phenotype to distinguish broomcorn and foxtail millet.

Some archaeologists and archaeobotanists have investigated the seed remains of broomcorn and foxtail millet discovered from archaeological sites in eastern Asia or the Middle East and established criteria to distinguish the caryopses of these two millets (e.g., Nesbitt and Summers 1988; Liu and Kong 2004; Fuller and Zhang 2007). Three features were identified. The first is caryopsis shape. Grains of broomcorn millet have a pointed distal end and relatively blunt proximal end, whereas grains of foxtail millet are gently rounded at both ends (Nesbitt and Summers 1988). The second criterion is the embryo notch. The embryo notch is short and wide for broomcorn millet but longer and more narrow for foxtail millet (Knorzer 1971; Nesbitt and Summers 1988). The third criterion is the morphology of the lemma and palea. Carbonized husk fragments of broomcorn millet are smooth and glossy, and foxtail millet fragments vary from finely rugose to punctuate (Knorzer 1971; Nesbitt and Summers 1988). Several features, such as the length-to-width ratio of the grain and surface sculpture of the lemma (especially the upper lemma), have been used to distinguish foxtail millet from its wild relatives (Musil 1963; Renfrew 1973; Nasu et al. 2007).

To provide numeric information about the embryo notch, we performed further analysis. The embryos contained high-quantity protein and lipids and thus would easily undergo microorganism degradation with only the cavity left. We removed the lemma, palea and embryo from seeds of modern millet, the landrace grown in the aboriginal village, and the de-embryonated seed looked quite similar to the unearthed seeds (Fig. 2). We then measured the angle, depth percentage and width percentage of the embryo notch for both modern and ancient millet seeds (Table 1). The angle was larger, by about 20°, for both ancient and modern broomcorn millet (average 104°) than ancient and modern foxtail millet (average 81°). The depth ratio was about 40% for the broomcorn millet and 60% for foxtail millet. The embryo notch width ratio was greater for both ancient and modern broomcorn than foxtail millet, about 50 vs. 30%. Thus, the morphology of the embryo notch indeed provided a good index to distinguish the unearthed seeds of broomcorn and foxtail millet in that the embryo notch angle was larger for broomcorn millet than foxtail millet by about 20°. The embryo notch depth ratio for the foxtail millet was greater than one half, and that of the broomcorn millet was less than one half. The embryo notch width ratio for broomcorn millet was about one half, and that of foxtail millet was only about one-third.

**Table 1:** Millet seed features

	Angle (°)	Depth (%)	Width (%)
Broomcorn millet			
Ancient	111.6 ± 11.3	37 ± 6	53 ± 7
Modern	96.5 ± 25.8	43 ± 8	48 ± 5
Foxtail millet			
Ancient	88.8 ± 25.6	66 ± 7	31 ± 6
Modern	72.6 ± 22.9	55 ± 11	33 ± 7

The angle, depth percentage and width percentage of the embryo notch for modern and ancient carbonized seeds of broomcorn millet (*Panicum miliaceum*) and foxtail millet (*Setaria italica*). Thirty seeds each kind were used for measurement

**Fig. 6:**
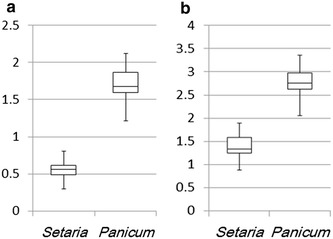
Estimated **a** volume and **b** area index per seed of carbonized millet seeds. **a** In total, 30 broomcorn millet (*Panicum miliaceum*) and 98 foxtail millet (*Setaria italica*) seeds were used for estimations. Length, width and thickness were measured by using a digital ruler. The *Y-axis* is for seed volume and the unit is mm^−3^. **b** The area index per seed of the two millet seed types was estimated from the scanned images of 50 seeds each. The *Y-axis* is for seed area index and the unit is mm^−2^. Data are *box*-and-*whisker plots* (median and interquartile range, Schmuller 2013) were used for illustration

We further analyzed the volume of unearthed seeds. A digital ruler was used to carefully measure the width, length and thickness so as to not crush the fragile ancient seeds. The mean volume per seeds was 0.55 ± 0.28 and 1.71 ± 0.19 mm^3^ for foxtail and broomcorn millet, respectively (Fig. 6a shows median [interquartile range] values). Because measuring many fragile ancient seeds was not convenient, we tried to monitor the seed area in scanned images, then estimated the area index by using ImageJ. The mean area index per seed was 1.32 ± 0.13 and 2.77 ± 0.01 mm^2^ for foxtail and broomcorn millet, respectively (Fig. 6b shows median [interquartile range] values). Because both the volume and area were larger for broomcorn millet than foxtail millet, either number may be used as an index to distinguish them. However, because the largest foxtail millet and smallest broomcorn millet seeds may overlap (Fig. 6), we need to confirm these seeds by using other characters such as the embryo notch mentioned above. Thus, we describe here a quick method to separate broomcorn and foxtail millet in a collection of unearthed small grains.

### Broomcorn millet (*P. miliaceum*) seeds at NKLE

**Fig. 7:**
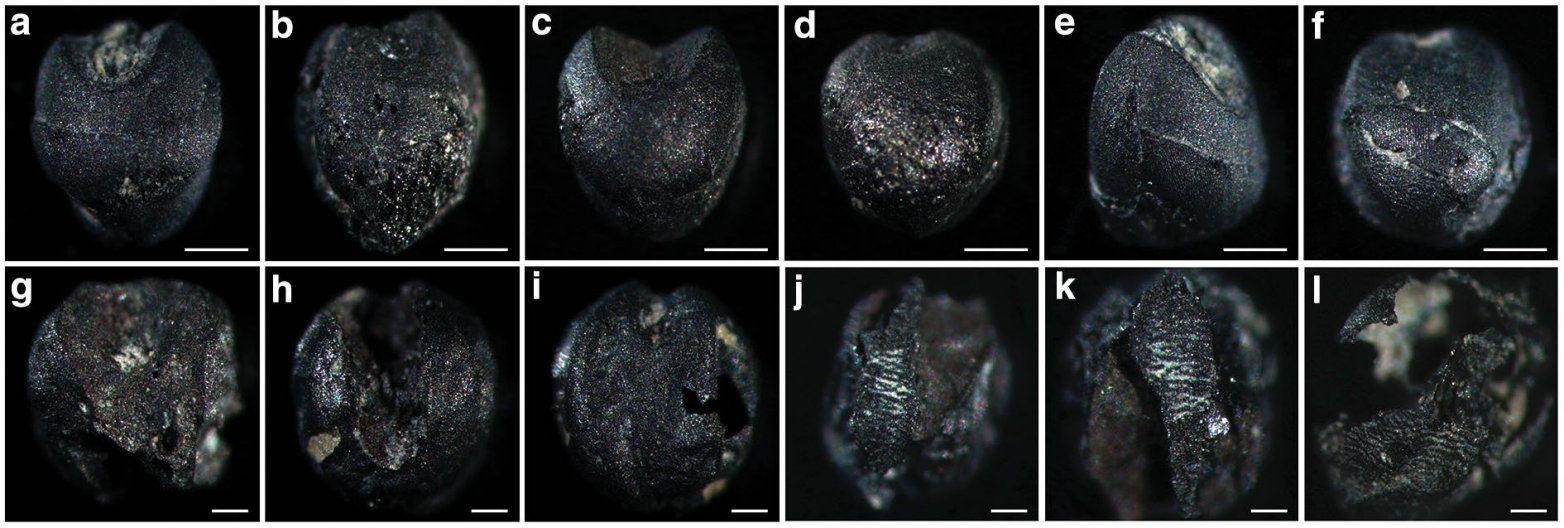
Stereomicroscopy of archaeological seed remains. **a**–**f** *P. miliaceum*; **g**–**i** *S. italica*; **j**–**l** *S. glauca*. **a**, **c**, **g**, **h** *ventral view*; **b**, **d**, **f**, **i**, **j**, **k**, **l** *dorsal view*; **e** *lateral view*. *Scale bars* **a**–**f** 0.5 mm, **g**–**l** 0.2 mm

Because the unearthed seeds present at NKLE had been buried for thousands of years, the materials were fragile. We compared the dorsal, ventral and lateral views of current and carbonated seeds by stereomicroscopy (Figs. 3, 7). Most of the millets are members of the tribe Paniceae, so we first examined one species, broomcorn millet. The seeds of the modern variety (Fig. 3a, b) and those from the Smangus aboriginal village (Fig. 3c, d) were examined. The ventral surface of the modern seeds was smooth and glossy, with palea in the center and two partial lemma at both sides (Fig. 3a, c). The whole dorsal surface was smooth and glossy (Fig. 3b, d). The distal end was pointed (Fig. 3a–d). In addition, the mean seed size was larger for broomcorn than foxtail millet (Fig. 3a–d vs. e–l).

**Fig. 8:**
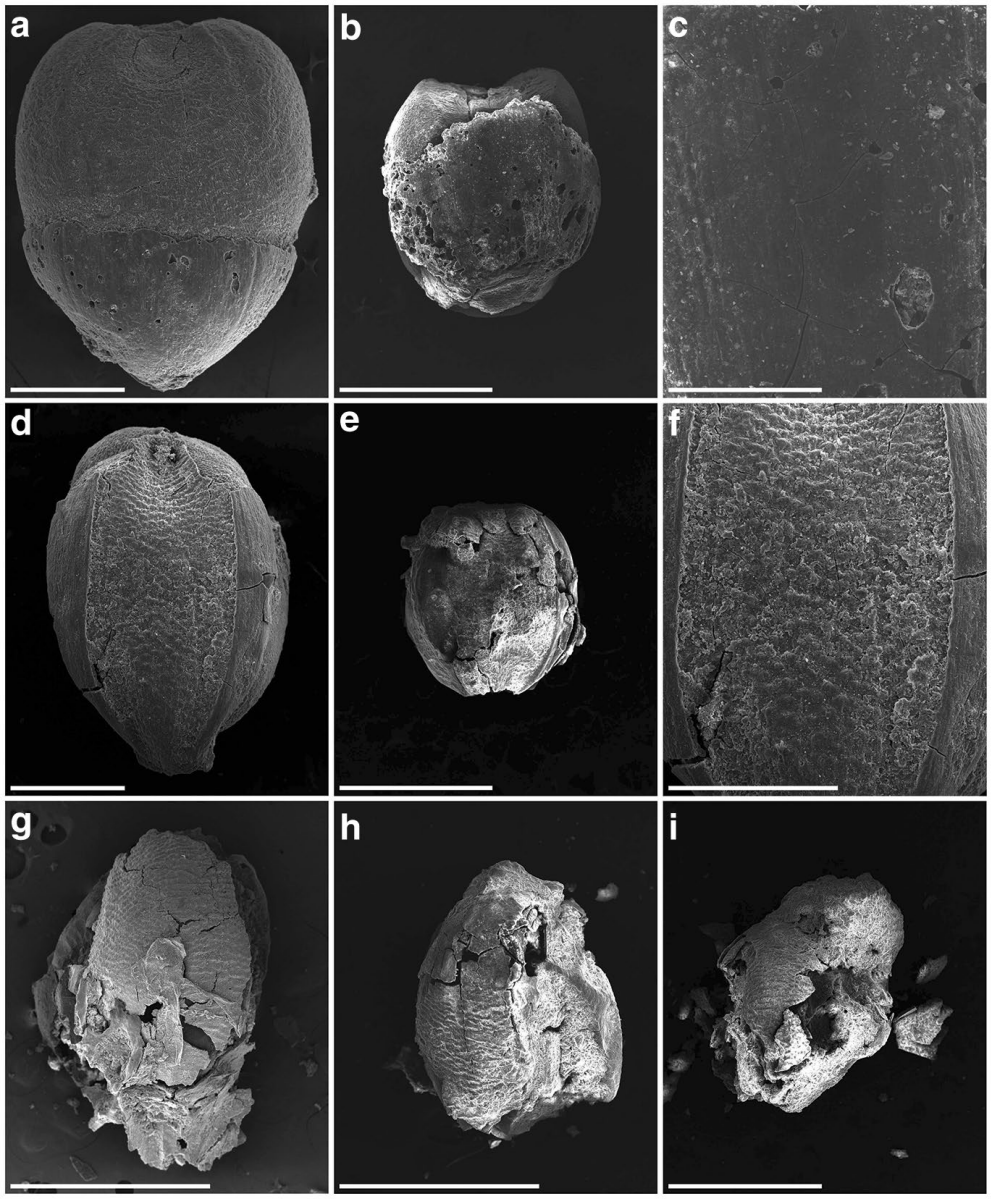
Scanning electron microscopy of archaeological seed remains. **a**–**c** *P. miliaceum*; **d**–**f** *S. italica*; **g**–**i** *S. glauca*. *Scale bars* **a**, **d** 0.5 mm; **b**, **e** 1 mm; **c**, **f** 0.2 mm; **g**–**i** 1 mm

Some unearthed grains featured the upper lemma and palea with the caryopsis inside, and some were naked grains. Figure 7a–f shows a close-up view of four millet unearthed seeds. Panels a and b are the same seeds, as are panels c and d. Most do not contain the palea structure, but panel f shows a small piece remaining. Since the structure is smooth and glossy, the seed may be broomcorn millet. The embryo groove of these seeds is wide (Fig. 7a–c), which again illustrates broomcorn millet seed. Panels a to d also show the pointed distal end of these seeds. As well, these seeds (panels a to f) are larger than those in panels g to l (see scale bars). SEM was used to further reveal the detailed structure of the unearthed seeds (Fig. 8). About one-third of the husk is still present in the seed (Fig. 8a). The palea locates at the center and is smooth and glossy, with two partial lemma at both sides. Another seed (Fig. 8b) contains a partial palea (zoom-in image in Fig. 8c); again the palea surface is smooth and glossy. All these characteristics fulfill the three criteria for broomcorn millet. Therefore, we confirmed that broomcorn millet was present at NKLE.

### *S. italica* and *S. glauca* seeds at NKLE

**Fig. 9:**
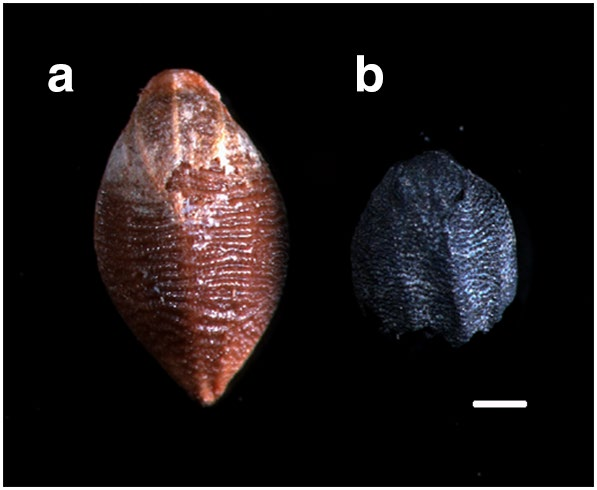
Stereomicroscopy of yellow foxtail seed. **a** Modern yellow foxtail seed, **b** partial archaeological seed remains at NKLE site. *Scale bar* 0.2 mm

The unearthed seeds were classified by their size and appearance at NKLE. The broomcorn and foxtail millet seeds were always classified in the same category. We noted another category of seeds in the plant remains. Counting the exact seed number was difficult because most were broken and the estimated total amount was about 10,000 seeds. Figure 9 illustrates one carbonized seed along with a seed from a modern *S. glauca* (also known as yellow foxtail, golden foxtail, or wild millet). Both seeds have large-diameter papillae arranged on conspicuous horizontal ridges. Because of the similarity of the seed appearance, the unearthed seed might be yellow foxtail. We then compared these seeds with other *Setaria* species, illustrated in the work by Nasu et al. (2007), who showed images of many other *Setaria* seeds that we could not find in Taiwan. The authors suggested that *Setaria* taxa were divided into slender and round groups on the basis of mean length-to-width ratios. *S. viridis*, another wild *Setaria* species, was the most slender. In addition, the surface of palea was grid-shaped. Using these morphological characters, we suggest only *S. glauca* but not *S. viridis* seed at the NKLE site.

Two kinds of the present *Setaria* seeds are shown in Fig. 3 (*S. italica* in e to h and *S. glauca* in i to l). The ratio of grain length to breadth for the two kinds of seed differs, with yellow foxtail having a slender shape and foxtail millet a round shape. Figure 7e and f are the same seeds, as are g and h, i and j, and k and l, one from the ventral side and the other the dorsal side. Yellow foxtail shows distinct horizontal ridges on the lemma at the dorsal sides (Fig. 3j, l). Thus, these characteristics provide reliable ways of separating the two species. Most of the smaller unearthed grains were not intact and had punctated husk fragments (Fig. 7g–l), especially on SEM (Fig. 8e–i). Because the sample chamber has to be in vacuum status for SEM observation, many of the *Setaria* seeds were crushed.

The unearthed millet seeds show two types: three (Fig. 7g–i) with a round shape and another three (Fig. 7j–l) with a slender shape. In addition, the three slender ones have the typical horizontal ridges on the upper lemma, the hallmark of yellow foxtail. Thus, the three round ones are foxtail millet and the other three are yellow foxtail. The SEM images of Fig. 8g–i illustrate that they should be seeds of yellow foxtail because of the horizontal ridges on the upper lemma (all dorsal sides). Figure 8d to f illustrates that these carbonized seeds are foxtail millet because of (1) the round caryopsis shape and (2) the punctate rather than smooth palea surface.

Both foxtail and yellow foxtail are still commonly consumed in India and Southern Asia (Sakamoto 1987; Kimata et al. 2000), the NKLE residents might have also consumed yellow foxtail seeds thousand years ago. More than 10,000 yellow foxtail seeds were unearthed, indicating these plants, a wild species, were abundant near the NKLE village. According to the records for aboriginal agriculture in the Ching Dynasty, rice, broomcorn and foxtail millet but not yellow foxtail were cultivated in the village (Chiang 1694). Yellow foxtail grows well in the countryside these days, according to a herbarium record in Taiwan (http://hast.sinica.edu.tw/). Thus, yellow foxtail may have been common near the NKLE village thousands of years ago. Even though the seeds were from wild species, the residents may have harvested and consumed them. Thus, their presence has implications for human subsistence practices.

### Mixed farming of millet and rice at NKLE

Were the rice and millet grain in NKLE from domesticated crops or wild species? That is, we wondered whether a farming system existed. The development of the rice grain has a unique characteristic. The maturation degree of florets differs in one single panicle (i.e., the flowering time of each floret in one panicle differs from the earliest to the latest for about 10 days), and the use of shell knives in NKLE indicated that these seeds did not shatter on maturation. If the seed of the rice and two millets did not shatter, they were not wild species. In addition, the site contained 8387 and 110,860 rice and millet grain, respectively, which indicates relatively extensive cultivation of both rice and millet. Thus, we suggest the existence of rice and mixed millet agriculture about 5000 years ago in NKLE, with millet species including broomcorn and foxtail millet. There was no field ridge or clayed soil, the index of paddy field practice (Tsang and Li 2015; Li 2013). In addition, no barnyard grass seed was found in the plant remains, again indicating an upland practice (Yang et al. 2015). Thus, the field was an upland practice. Mixed farming of millet and rice was also evident in central–north China at several unearthed sites. For instance, early mixed farming of millet and rice 7800 years ago (Peiligang Culture) was discovered in the middle Yellow River Region (Zhang et al. 2012); rice, broomcorn millet and foxtail millet were present. Further analysis will be necessary to determine where the NKLE residents and crops came from.

## Conclusions

We identified two domesticated millet, broomcorn millet (*Panicum miliaceum*) and foxtail millet (*Setaria italica*), as well as one wild millet species, yellow foxtail (*Setaria glauca*), at the NKLE archeological site, which, along with the previously identified rice remains, indicates that four kinds of cereals were consumed about 5000 BP during the Tapenkeng Culture. All four species were abundant in the unearthed site (i.e., 8717; 33,000; 77,000; and about 10,000 for rice, broomcorn millet, foxtail millet, and yellow foxtail seeds, respectively). Such high quantity indicates that these seeds were consumed thousands of years ago, in addition to food from fishing and hunting. The mixed millet–rice farming might have played an important role in the early civilization period. Our results indicate that NKLE was an important location for early hunter-gatherer-fisher-farmers about 5000 years ago in southern Taiwan.
